# Supplementary material for: CaptureSeq: Hybridization-Based Enrichment of cpn60 Gene Fragments Reveals the Community Structures of Synthetic and Natural Microbial Ecosystems
Source: Microorganisms. 2021 Apr 13;9(4):816. doi: 10.3390/microorganisms9040816 (PMC8069376; doi:10.3390/microorganisms9040816)
Supplement: Supplementary file 1 [file microorganisms-09-00816-s001.zip › Supplemental Table S2-MVP targets.docx]

|  | Amplicon | | | CaptureSeq | | |
| --- | --- | --- | --- | --- | --- | --- |
|  | High | Medium | Low | High | Medium | Low |
| *Atopobium vaginae* | 171 | 133 | 10 | 943 | 87 | 10 |
| *Bifidobacterium bifidum* | 10 | 9 | 14 | 827 | 15 | 29 |
| *Bifidobacterium infantis* | 95 | 57 | 20 | 527 | 35 | 4 |
| *Gardnerella vaginalis* | 1,096 | 974 | 120 | 1,369 | 92 | 11 |
| *Lactobacillus* sp. N27 | 193 | 86 | 11 | 295 | 15 | 4 |
| *Lactobacillus iners* | 859 | 616 | 94 | 741 | 66 | 8 |
| *Lactobacillus crispatus* | 684 | 356 | 60 | 731 | 55 | 15 |
| *Lactobacillus jensenii* | 2,090 | 1,373 | 143 | 1,020 | 92 | 11 |
| *Lactobacillus vaginalis* | 529 | 281 | 33 | 946 | 79 | 17 |
| *Lactobacillus gasseri* | 2,330 | 1,079 | 135 | 827 | 69 | 12 |
| *Lactobacillus salivarius* | 1,554 | 1,254 | 126 | 874 | 63 | 2 |
| *Lactobacillus* sp. L6 | 652 | 324 | 39 | 803 | 65 | 10 |
| *Lactobacillus* sp. N19 | 2,756 | 1,864 | 229 | 889 | 83 | 9 |
| *Lactobacillus johnsonii* | 426 | 317 | 43 | 297 | 20 | 3 |
| *Lactobacillus* sp. N20 | 4,142 | 3,382 | 416 | 2,466 | 244 | 29 |
| *Peptostreptococcus anaerobius* | 2,468 | 1,681 | 107 | 1,416 | 124 | 18 |
| *Prevotella bivia* | 817 | 357 | 50 | 959 | 83 | 11 |
| *Streptococcus gallolyticus* | 3,789 | 2,153 | 173 | 1,010 | 89 | 12 |
| *Streptococcus lutetiensis* | 1,048 | 623 | 46 | 769 | 66 | 10 |
| *Lactobacillus plantarum* | 1,291 | 729 | 126 | 1,332 | 120 | 18 |

**Table S2.** Sequencing read abundances mapping to each taxonomic cluster for seed wash samples spiked with a synthetic community consisting of 20 *cpn60* UT plasmids in 10-fold decreasing dilutions (high, medium, low) as described in the text. Amplicon and CaptureSeq data were downsampled to 30,091 and 506,247 sequencing reads respectively and mapped to a reference dataset consisting of the *cpn60* UT sequences of the 20 bacteria in the panel.
