# Supplementary material for: CaptureSeq: Hybridization-Based Enrichment of cpn60 Gene Fragments Reveals the Community Structures of Synthetic and Natural Microbial Ecosystems
Source: Microorganisms. 2021 Apr 13;9(4):816. doi: 10.3390/microorganisms9040816 (PMC8069376; doi:10.3390/microorganisms9040816)
Supplement: Supplementary file 1 [file microorganisms-09-00816-s001.zip › Supplemental Table S3-read abundances.docx]

| Profiling method | Treatment  (mg kg^-1^) | Plot | Sequencing  reads | Reference Mapped reads |
| --- | --- | --- | --- | --- |
| Amplicon | 0 | 1 | 254,145 | 94.9% |
|  | 0 | 7 | 34,221 | 95.8% |
|  | 0 | 12 | 39,603 | 94.5% |
|  | 10 | 4 | 161,181 | 94.3% |
|  | 10 | 8 | 65,299 | 95.1% |
|  | 10 | 11 | 29,014 | 94.4% |
| CaptureSeq | 0 | 1 | 1,226,019 | 16.4% |
|  | 0 | 7 | 1,172,125 | 18.1% |
|  | 0 | 12 | 1,158,656 | 15.9% |
|  | 10 | 4 | 1,025,680 | 17.0% |
|  | 10 | 8 | 827,129 | 16.4% |
|  | 10 | 11 | 765,101 | 16.7% |
| Shotgun metagenomic | 0 | 1 | 6,116,268 | 0.070% |
|  | 0 | 7 | 7,306,386 | 0.071% |
|  | 0 | 12 | 6,953,220 | 0.070% |
|  | 10 | 4 | 4,232,651 | 0.067% |
|  | 10 | 8 | 4,794,243 | 0.067% |
|  | 10 | 11 | 4,287,559 | 0.069% |

**Table S3.** Read numbers (total and proportion mapping to the *cpn60* reference dataset) obtained using *cpn60* amplicon, CaptureSeq, and shotgun metagnomic profiling methods on soil samples.
