# Supplementary figures and images for: CaptureSeq: Hybridization-Based Enrichment of cpn60 Gene Fragments Reveals the Community Structures of Synthetic and Natural Microbial Ecosystems
Source: Microorganisms. 2021 Apr 13;9(4):816. doi: 10.3390/microorganisms9040816 (PMC8069376; doi:10.3390/microorganisms9040816)

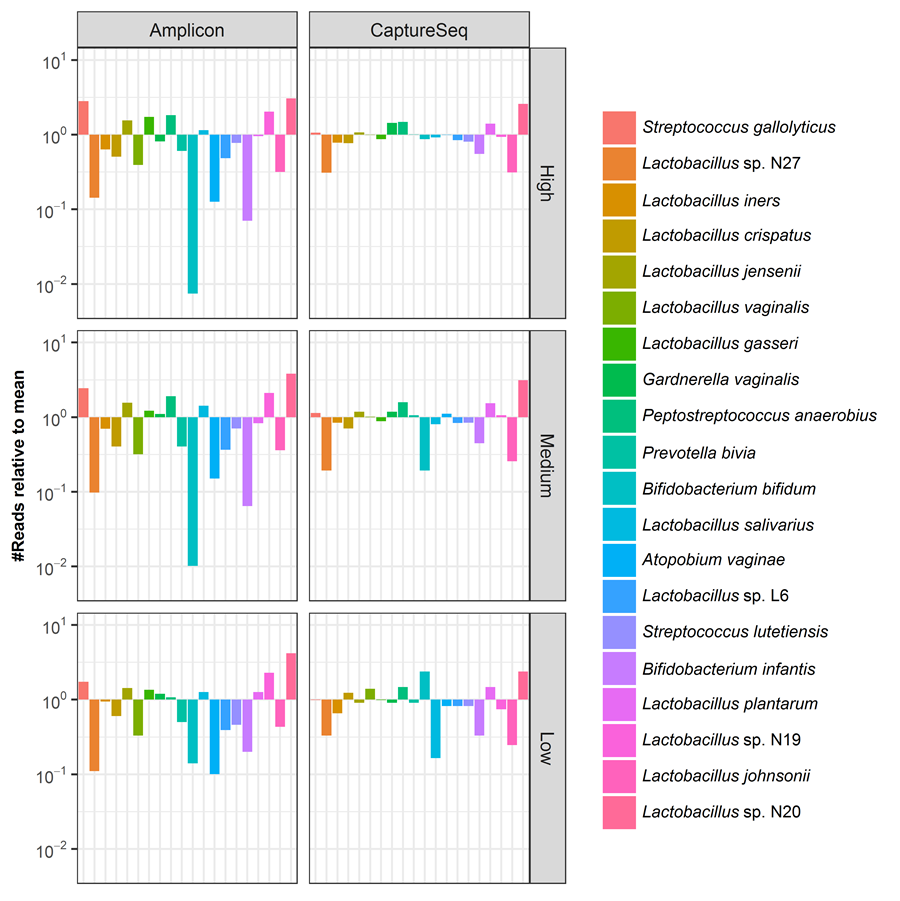

Supplement: Supplementary file 1 [file microorganisms-09-00816-s001.zip › Supplemental Figure S1.tif]
